# Severe Pulmonary Arteriopathy Is Associated with Persistent Hypoxemia after Pulmonary Endarterectomy in Chronic Thromboembolic Pulmonary Hypertension

**DOI:** 10.1371/journal.pone.0161827

**Published:** 2016-08-29

**Authors:** Takayuki Jujo, Nobuhiro Tanabe, Seiichiro Sakao, Hatsue Ishibashi-Ueda, Keiichi Ishida, Akira Naito, Fumiaki Kato, Takao Takeuchi, Ayumi Sekine, Rintaro Nishimura, Toshihiko Sugiura, Ayako Shigeta, Masahisa Masuda, Koichiro Tatsumi

**Affiliations:** 1 Department of Respirology (B2), Graduate School of Medicine, Chiba University, 1-8-1, Inohana, Chuo-Ku, Chiba City, 260–8670, Japan; 2 Department of Advanced Medicine in Pulmonary Hypertension, Graduate School of Medicine, Chiba University, 1-8-1 Inohana, Chuo-Ku, Chiba City, 260–8670, Japan; 3 Department of Pathology, National Cerebral and Cardiovascular Center, 5-7-1, Fujishiro-Dai, Suita City, Osaka, 565–8565, Japan; 4 Department of Cardiovascular Surgery, Graduate School of Medicine, Chiba University, 1-8-1 Inohana, Chuo-Ku, Chiba City, 260–8670, Japan; 5 Department of Cardiovascular Surgery, Chiba Medical Center, National Hospital Organization, 4-1-2, Tsubakimori, Chuo-ku, Chiba City, 260–8606, Japan; Professor, JAPAN

## Abstract

**Background:**

Chronic thromboembolic pulmonary hypertension (CTEPH) is characterized by occlusion of pulmonary arteries by organized chronic thrombi. Persistent hypoxemia and residual pulmonary hypertension (PH) following successful pulmonary endarterectomy (PEA) are clinically important problems; however, the underlying mechanisms remain unclear. We have previously reported that residual PH is closely related to severe pulmonary vascular remodeling and hypothesize that this arteriopathy might also be involved in impaired gas exchange. The purpose of this study was to evaluate the association between hypoxemia and pulmonary arteriopathy after PEA.

**Methods and Results:**

Between December 2011 and November 2014, 23 CTEPH patients underwent PEA and lung biopsy. The extent of pulmonary arteriopathy was quantified pathologically in lung biopsy specimens. We then analyzed the relationship between the severity of pulmonary arteriopathy and gas exchange after PEA. We observed that the severity of pulmonary arteriopathy was negatively correlated with postoperative and follow-up PaO_2_ (postoperative PaO_2_: r = -0.73, p = 0.0004; follow-up PaO_2_: r = -0.66, p = 0.001), but not with preoperative PaO_2_ (r = -0.373, p = 0.08). Multivariate analysis revealed that the obstruction ratio and patient age were determinants of PaO_2_ one month after PEA (R^2^ = 0.651, p = 0.00009). Furthermore, the obstruction ratio and improvement of pulmonary vascular resistance were determinants of PaO_2_ at follow-up (R^2^ = 0.545, p = 0.0002). Severe pulmonary arteriopathy might increase the alveolar-arterial oxygen difference and impair diffusion capacity, resulting in hypoxemia following PEA.

**Conclusion:**

The severity of pulmonary arteriopathy was closely associated with postoperative and follow-up hypoxemia.

## Introduction

Chronic thromboembolic pulmonary hypertension (CTEPH) is characterized by occlusion of pulmonary arteries by organized chronic thrombi [1,2]. It is thought that inappropriate resolution of acute pulmonary embolism results in the formation of persistent chronic thrombi leading to pulmonary hypertension [3]. Pulmonary endarterectomy (PEA) is a surgical procedure in which the organized pulmonary thrombi are removed. PEA has been shown to improve hemodynamics, cardiac function, and the six-minute walk distance in patients with CTEPH [4–6]. However, following PEA, many patients experience persistent hypoxemia and residual pulmonary hypertension (PH). Hypoxemia and residual PH have been observed in 50–60% [7] and 5–35% [8] of CTEPH patients, respectively, following successful PEA and are closely related to poor functional capacity (NYHA functional class III-IV) [9].

**Fig 1:**
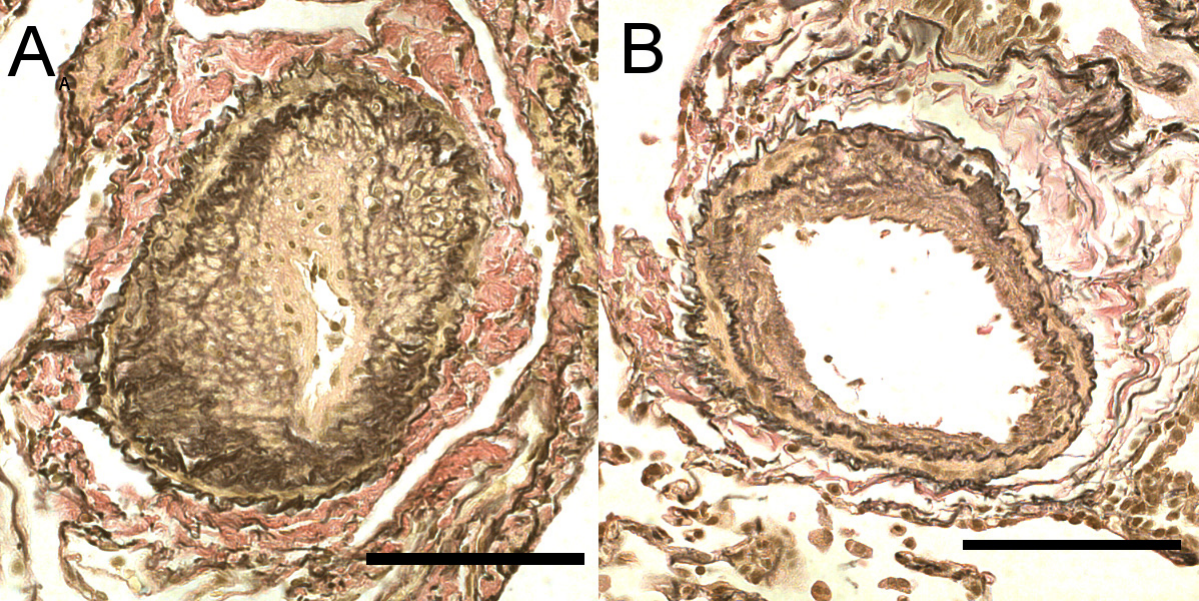
Pulmonary arteriopathy in biopsied lung tissues. Severe pulmonary arteriopathy in the high-obstruction group (A). Pulmonary arteriopathy was composed of severe fibrous intimal thickening, moderate medial hypertrophy, and lumen stenosis. The low-obstruction group (B) demonstrated mild pulmonary intimal thickening and medial hypertrophy.

Ventilation-perfusion mismatch has been postulated as a major contributor to hypoxemia in CTEPH patients [10,11]. However, the anatomical location and the pathophysiology underlying this finding remain unclear. We recently reported that residual PH was associated with severe pulmonary arteriopathy [12] and hypothesized that severe pulmonary vascular remodeling might also be involved in impaired gas exchange (Fig 1). Thus, the purpose of this study was to evaluate the relationship between pulmonary arteriopathy and gas exchange following PEA.

## Methods

### Ethics statement

This study was approved by the Ethics Committee of Chiba University (approval number: 1221). Written informed consent for inclusion in this study was obtained from all patients.

### Subjects

Thirty CTEPH patients underwent PEA between December 2011 and November 2014 in our institutions. Lung biopsy was performed in 25 patients. Two of these patients were excluded from the study because the specimens were too small to yield adequate slices for examination. Thus, lung tissues derived from 23 patients were evaluated in this study. All subjects were examined and diagnosed with CTEPH at Chiba University Hospital, which is located about 20 meters above sea level. The diagnosis was confirmed by right heart catheterization (RHC), pulmonary angiography (PAG), lung perfusion scans, and enhanced computed tomography (CT). The criteria for CTEPH diagnosis were as follows: 1) elevation of mean pulmonary arterial pressure (mean Ppa ≥ 25 mmHg) with normal pulmonary arterial wedge pressure (PAWP); 2) symptoms for more than six months; 3) persistent pulmonary embolism confirmed by lung perfusion scans, CT scans and PAGs. General examinations, respiratory function tests, CT scans, RHC, and PAG were performed during the preoperative, postoperative (about one month after surgery), and follow-up periods. Follow-up evaluation was performed 13.1±2.4 months after PEA. For one female patient, postoperative and follow-up CT scans were not obtained due to an anaphylactic reaction to contrast medium. This patient also refused postoperative RHC.

### RHC

RHC was performed in a similar method as described in our previous report [12]. Briefly, Ppa and PAWP were measured using a 7.5-Fr Swan-Ganz catheter (Edwards Lifesciences, USA). Cardiac index (CI) was calculated from cardiac output (CO) measured by thermodilution. Pulmonary vascular resistance (PVR) was calculated according to the following formula:

$$\mathrm{PVR}\;\left(dyne\cdot sec\cdot {cm}^{-5}\right)=\frac{mean\;Ppa-PAWP}{CO}\times 80$$

### Blood gas analysis

Blood samples from the pulmonary artery (mixed venous blood) and a peripheral systemic artery were obtained at the time of pulmonary pressure measurement, while each patient was breathing room air. The partial pressures of oxygen and carbon dioxide in systemic blood (PaO_2_ and PaCO_2_, respectively) and the partial pressure of oxygen in mixed venous blood (PvO_2_) were directly measured by a blood gas analyzer (ABL-555, Radiometer Medical Aps, Copenhagen, Denmark). The alveolar-arterial oxygen difference (A-aDO_2_) was calculated according to the following formula [13]:

$${A-aDO}_{2}\left(mmHg\right)=\left[F_{I}O_{2}\left(P_{atm}-P_{vapor}\right)-\frac{P_{a}{CO}_{2}}{R}+F_{I}O_{2}\times P_{a}{CO}_{2}\times \frac{1-R}{R}\right]-P_{a}O_{2}$$

in which F_I_O_2_ is the fraction of inspired oxygen (0.209), P_atm_ is the atmospheric pressure (760 mmHg), P_vapor_ is the saturated water vapor pressure (47 mmHg), and R is the respiratory quotient (0.83). Two blood samples obtained from two female patients during the postoperative period were excluded because they showed a negative A-aDO_2_, which was considered a technical error resulting from a lack of oxygen washout before the measurement.

### PEA

PEA was performed by Drs. K.I and M.M at Chiba University Hospital and the Chiba Medical Center. The indications for PEA were similar to those described in our previous report [12]: 1) mean Ppa > 30 mmHg, 2) PVR > 300 dyne·sec·cm^-5^, 3) WHO functional class ≥ 2, 4) technically operable, and 5) no significant comorbidity. In patients with mild PH (mean Ppa 25–30 mmHg), PEA was performed only if the patient wished to undergo the procedure after the risks and benefits had been fully explained.

### Lung biopsy and sample preparation

Lung biopsy was performed during PEA. The lung biopsy technique and pathological evaluation were similar to our previous report [12]. Lung tissue was resected from the right middle lobe or left lingular segment. Lung biopsy was limited to a single site for ethical and safety reasons. The selection of the areas for biopsy was left to the surgeon’s discretion. The biopsy sites were selected by considering safety, and the selection was unrelated to removal of thrombi. Samples were fixed in 10% buffered formalin and embedded in paraffin. More than 20 sections obtained at intervals of at least 20 μm were prepared for each patient. A total of 555 slices were prepared. The sections were stained with Elastica van Gieson stain for quantification.

### Pathological quantification of pulmonary arteriopathy

Pathological quantification of pulmonary arteriopathy was performed according to the methods described in our previous report [12]. Details are shown in S1 Fig. The obstruction ratio of a pulmonary artery was defined as the ratio of the luminal and medial area to the vascular area; the obstruction ratio was calculated for each pulmonary artery using Image J software (version 1.45). Pathological findings were reviewed by a trained pathologist and a pulmonologist. The mean obstruction ratio for each patient was defined as the average value of all obstruction ratios in the tissue sample. The patients were divided into two groups, the high-obstruction group and the low-obstruction group, based on the median value of all mean obstruction ratios.

### Evaluation of segmental pulmonary thrombi

Chronic thrombi within segmental pulmonary arteries were quantified by enhanced CT scanning according to the modified methods of Qanadli [14]. CT scans were performed using the Aquilion ONE (Toshiba Medical, Tochigi, Japan). Contrast material consisting of 100 ml of 350 mg/ml of iodine was injected using a mechanical injector. CT scan images were obtained using a tube voltage of 120 kV and a scanning delay of 20–30 seconds. Each image was 0.5mm thick. Segmental pulmonary arteries were defined according to Boyden’s classification [15] and consisted of ten right-sided and eight left-sided segmental pulmonary arteries. Eighteen segmental pulmonary arteries were identified on each CT scan image and each segmental artery was evaluated over the entire series of images. Each segmental pulmonary artery was scored as follows: score 0: no thrombi; score 1: the artery was narrowed by chronic thrombi but contrast medium passed to distal areas; and score 2: the artery was obstructed by chronic thrombi and contrast medium did not pass to distal areas. The segmental obstruction index (SGOI) was calculated by the following equation:

$$SGOI=\frac{0\times n_{0}+1\times n_{1}+2\times n_{2}}{36}$$

in which n_0_, n_1_, and n_2_ are the numbers of segmental pulmonary arteries with score 0, score 1, and score 2, respectively. We also evaluated the presence or absence of thrombi in the biopsied area. Two segmental pulmonary arteries (A4, A5) in the biopsied side were scored. The segmental obstruction index at the biopsied site (SGOI_biopsy_) was calculated by the following equation:

$${SGOI}_{biopsy}=\frac{0\times n_{0}+1\times n_{1}+2\times n_{2}}{2}$$

Two investigators interpreted a total of 64 CT scan images in a blinded manner (T.J and A.N). The SGOI and SGOI_biopsy_ scores obtained by the two independent investigators were significantly correlated (SGOI: r = 0.917, p = 1.7×10^−26^; SGOI_biopsy_: r = 0.803, p = 1.4×10^−15^). To minimize bias, SGOI and SGOI_biopsy_ scores for each patient were defined as the average value between the two investigators.

### Statistical analysis

Continuous variables are reported as the mean ± SD unless otherwise indicated. The correlation between variables was determined by Spearman's rank correlation coefficient. Multiple comparisons between more than three groups were performed by one-way ANOVA with the adjustment of p-value by the Bonferroni method. Pairwise comparisons of time-dependent data were made by Repeated-Measures ANOVA with the adjustment of p-value by the Bonferroni method. The change of vasodilator usage rate was analyzed by McNemar's Chi-squared test. Simple and multiple linear regression analyses were used to determine the factors associated with PaO_2_. A p-value of < 0.05 was considered significant. All data were analyzed using EZR (ver. 1.29, Saitama Medical Center, Jichi Medical University, Saitama, Japan) [16].

## Results

### Clinical background of subjects

The 23 study patients included 4 males and 19 females. The mean age at diagnosis was 64.7 ± 8.1 years old. The mean period from symptom onset to PEA was 53.7 ± 52.8 months. Nineteen of 23 patients (82.6%) had a history of deep vein thrombosis. Lung tissues were biopsied from the right middle lobe of five patients and from the left lingular segment of eighteen patients. One patient developed sudden respiratory failure of unknown cause and died 15 days after PEA as described previously [12]. Including this patient, no complication related to the lung biopsies was observed.

Data from the three observational periods are shown in Table 1 and S1 Table. Following PEA, the burden of segmental organized thrombi was significantly reduced, and gas exchange parameters and hemodynamics improved. The follow-up PaO_2_ was significantly higher than the preoperative PaO_2_ (p = 0.0002). The postoperative PaO_2_ was higher than the preoperative PaO_2_, although the difference was not significant (p = 0.1).

**Table 1:** Patient characteristics.

	Preoperative	Postoperative	Follow-up
Segmental pulmonary thrombi	(n = 23)	(n = 21)	(n = 20)
SGOI	0.35 ± 0.15	0.22 ± 0.15*	0.21 ± 0.16*§
Hemodynamics	(n = 23)	(n = 21)	(n = 22)
Mean Ppa (mmHg)	45.1 ± 10.6	26.9 ± 9.3*	**28.1** ± 8.7*
PVR (dyne·sec·cm^-5^)	767 ± 299	335 ± 166*	374 ± 183*
CI (L/min/m^2^)	2.85 ± 0.79	3.15 ± 0.47	**2.83** ± **0.62§**
PAWP (mmHg)	7.9 ± 2.8	8.4 ± 3.3	9.0 ± 2.5
Blood gas analysis	(n = 23)	(n = 19)	(n = 22)
PaO_2_ (mmHg)	57.0 ± 10.1	61.5 ± 14.1	**71.3** ± 15.1*§
PaCO_2_ (mmHg)	37.2 ± 3.4	41.3 ± 3.1*	**41.5** ± 3.7*
PvO_2_ (mmHg)	33.8 ± 3.7	34.1 ± 3.3	**37.2** ± 3.2*§
A-aDO_2_ (mmHg)	48.8 ± 9.2	38.8 ± 14.8*	**29.7** ± 14.1*§
Respiratory function test	(n = 22)	(n = 21)	(n = 22)
%VC (%)	88.7 ± 12.4	78.1 ± 10.1*	**90.2 ± 13.9§**
FEV_1.0_/FVC (%)	76.3 ± 7.7	79.4 ± 7.4	76.1 ± 7.0
%DLCO/V_A_ (%)	80.4 ± 14.7	71.7 ± 11.2*	74.9 ± 15.9
Vasodilators	14/23	3/22*	7/22*
Shunt ratio by perfusion scan (%)	6.4±2.2 (n = 10)	6.5±0.8 (n = 5)	7.3±3.8 (n = 4)

SGOI: Segmental obstruction index, Ppa: pulmonary arterial pressure; PVR: pulmonary vascular resistance; CI: cardiac index; PAWP: pulmonary arterial wedge pressure; PaO_2_: arterial oxygen pressure; PaCO_2_: arterial carbon dioxide pressure; PvO_2_: oxygen pressure of mixed venous blood; A-aDO_2_: alveolar-arterial oxygen difference; %VC: vital capacity as the percent predicted; FEV_1.0_: forced expiratory volume (FEV) in 1 second; FVC: forced vital capacity; DL_CO_: diffusing capacity of carbon monoxide; V_A_: alveolar ventilation.

*: p<0.05 vs preoperative

**§**: p<0.05 vs postoperative.

### The relationship between pulmonary arteriopathy and PaO_2_ after PEA

**Fig 2:**
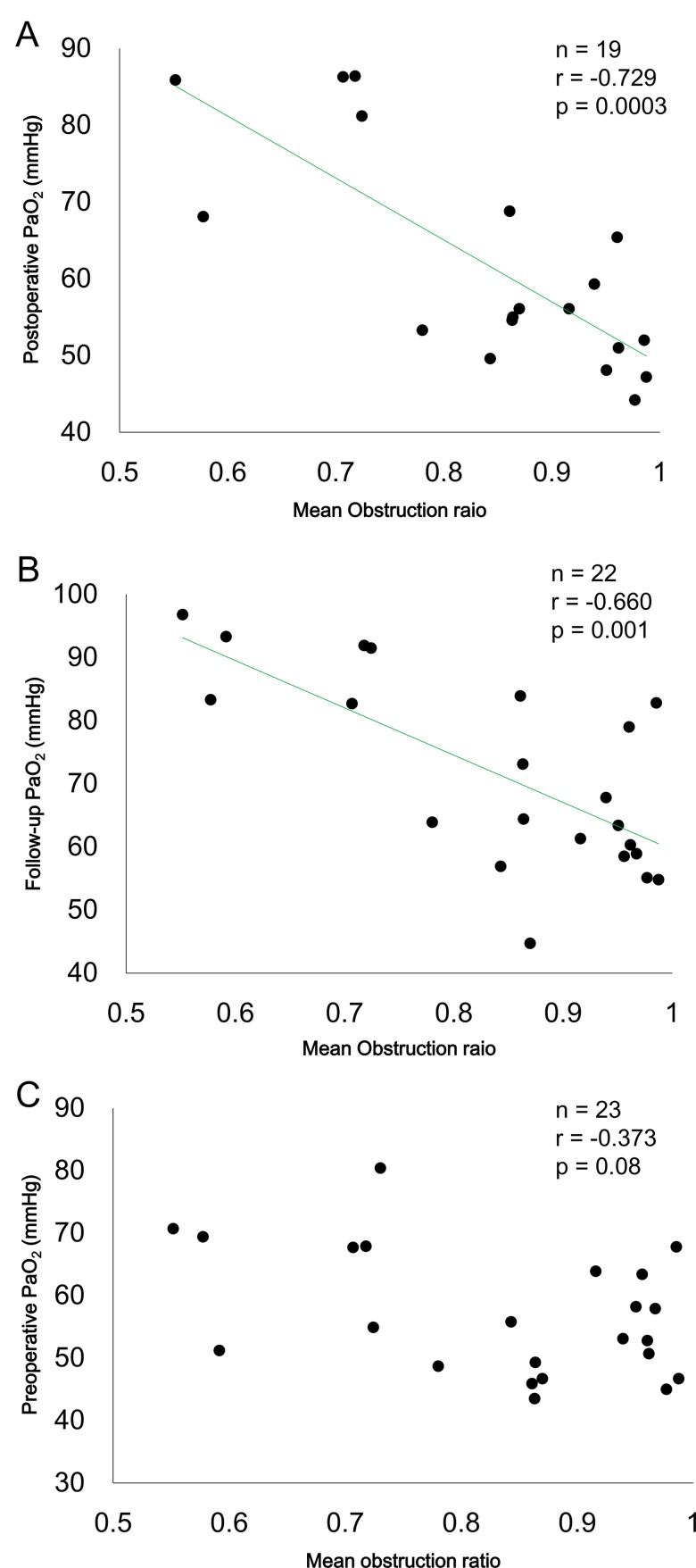
Correlation between mean obstruction ratio and PaO_2_. The mean obstruction ratio was negatively correlated with postoperative (A) and follow-up (B) PaO_2._ However, no correlation was observed between the mean obstruction ratio and preoperative PaO_2_ (C).

**Fig 3:**
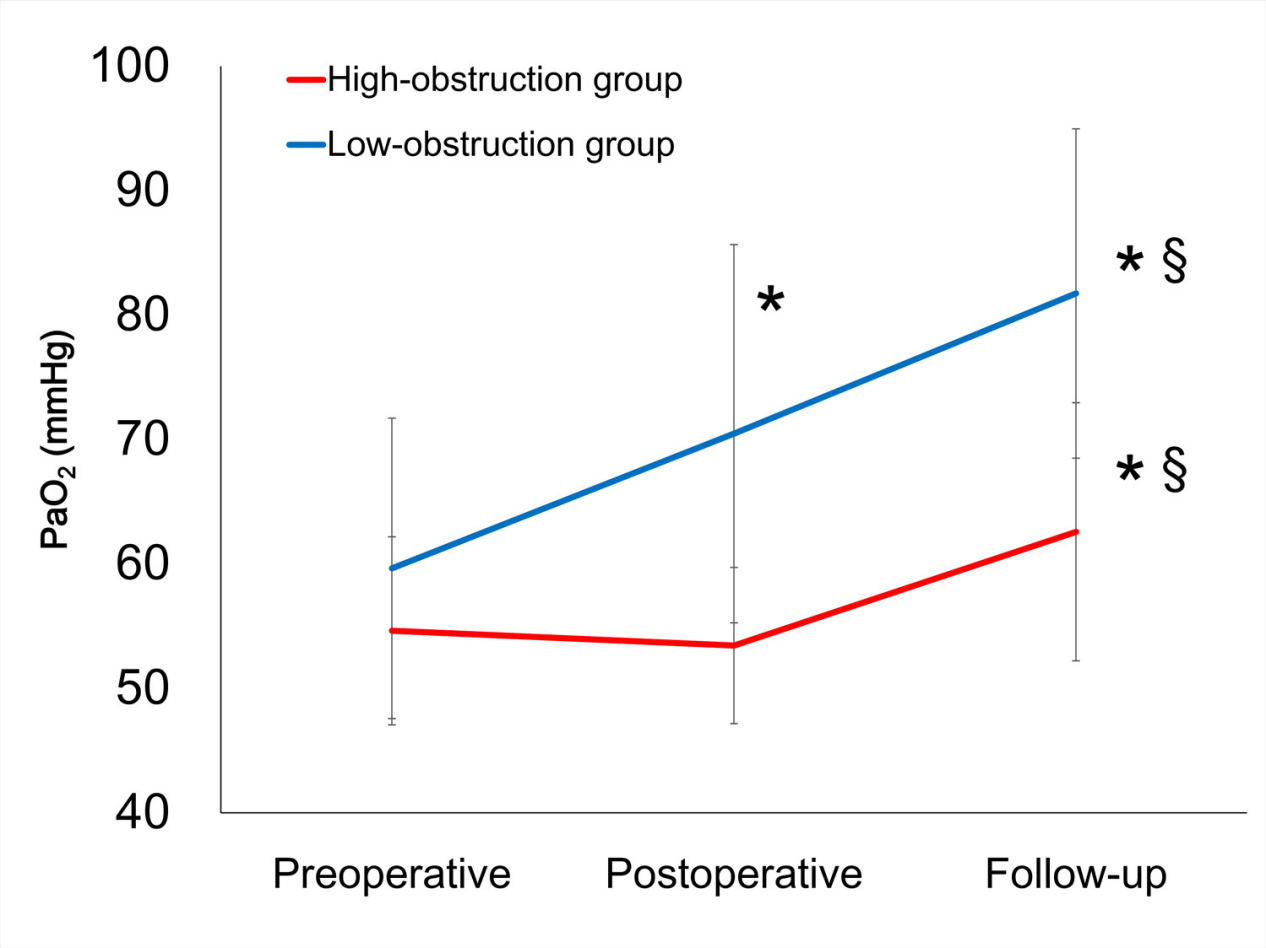
PaO_2_ in the high- and low-obstruction groups. The high obstruction group had lower PaO_2_ values than the low-obstruction group (p = 0.009, analyzed by repeated measures ANOVA). (*: p<0.05, vs preoperative data for each group; §: p<0.05, vs postoperative data for each group).

Quantitative data were obtained from 354 pulmonary muscular arteries in the 23 patients in this study. Pulmonary arteriopathy with intimal thickening and medial hypertrophy was observed in samples from all patients (Fig 1). The mean obstruction ratio was 0.838±0.139 and the median value of the mean obstruction ratios was 0.863933. No difference in the obstruction ratio was observed between biopsy sites (left: 0.837±0.146; right: 0.843±0.128, p = 0.9). The mean obstruction ratio was negatively correlated with the postoperative and follow-up PaO_2_ (Fig 2A and 2B), but was less correlated with the preoperative PaO_2_ (Fig 2C). The high obstruction group (mean obstruction ratio ≥0.863933) had lower PaO_2_ values than the low-obstruction group (mean obstruction ratio <0.863933) (p = 0.009, analyzed by repeated measures ANOVA, Fig 3).

### Segmental thrombi and gas exchange

The macroscopic classification of resected segmental organized thrombi is known as the “Jamieson’s classification” [4]. Samples were classified as follows, Type I, 9 cases; Type II, 9 cases, and Type III, 5 cases. The preoperative SGOI for each Jamieson’s classification was shown as follows, Type I, 0.44 ± 0.14; Type II, 0.33 ± 0.13; and Type III, 0.24 ± 0.14. There were no significant differences in SGOI among the three classes (p = 0.0501, analyzed by one-way ANOVA). The SGOI significantly decreased after PEA (preoperative vs. postoperative: p = 0.000003, preoperative vs. follow-up: p = 0.0000007, Table 1). Neither the preoperative SGOI nor the SGOI_biopsy_ correlated with the mean obstruction ratio (preoperative SGOI: r = 0.093, p = 0.7; preoperative SGOI_biopsy_: r = 0.143, p = 0.5). The SGOI did not correlate with PaO_2_ (preoperative: r = -0.024, p = 0.9; postoperative: r = -0.298, p = 0.2; follow-up: r = -0.298, p = 0.2) or A-aDO_2_ (preoperative: r = -0.134, p = 0.5; postoperative: r = 0.436, p = 0.06; follow-up: r = 0.320, p = 0.2). The SGOI weakly correlated with the postoperative %DL_CO_/V_A_ (r = -0.445, p = 0.04), but not with the preoperative and follow-up values (preoperative: r = 0.101, p = 0.7; follow-up: r = -0.090, p = 0.7).

### Pulmonary arteriopathy and hypoxemia after PEA

Patient age, preoperative PaO_2_, cardiac and respiratory function, reduction in PVR (%Δ PVR), SGOI, decrease in SGOI (Δ SGOI), and mean obstruction ratio were associated with PaO_2_. Multiple regression analysis using these factors revealed that the obstruction ratio correlated with both postoperative and follow-up PaO_2_ (Tables 2 and 3). Single regression analysis revealed that the mean obstruction ratio weakly correlated with preoperative PaO_2_ (standardized regression coefficient: -0.426, adjusted R-squared: 0.1421, p = 0.04,). The correlations between the other factors and preoperative PaO_2_ were poor (S2 Table).

**Table 2:** Univariate and multivariate analysis of variables associated with postoperative PaO_2_.

	Simple regression analysis		Multiple regression analysis			
	β	p-value	β	p-value	β	p-value
Age	-0.699 ± 0.164	0.0005	-0.345 **±** 0.184	0.08	-0.391 **±** 0.171	0.04*
Preoperative PaO_2_	0.620 ± 0.213	0.01	0.060 **±** 0.215	0.8		
Postoperative SGOI	-0.282 ± 0.236	0.2				
Δ SGOI	0.268 ± 0.223	0.2				
Postoperative CI	0.016 ± 0.238	0.945				
%Δ PVR	0.466 ± 0.215	0.04	0.128 **±** 0.175	0.5		
**Obstruction ratio**	**-0.797 ± 0.162**	**0.0001**	**-0.492 ± 0.203**	**0.03**	**-0.543 ± 0.182**	0.009*
Posoperative %VC	0.065 ± 0.253	0.8				
Postperative FEV_1.0_/FVC%	0.337 ± 0.220	0.1				
Postoperative %DLCO/V_A_	0.411 ± 0.217	0.08				

β = standardized regression coefficient ± standard error.

*Adjusted R^2^ = 0.651, p = 0.00009, See Table 1 for abbreviations.

**Table 3:** Univariate and multivariate analysis of variables associated with follow-up PaO_2_.

	Simple regression analysis		Multiple regression analysis			
	β	p-value	β	p-value	β	p-value
Age	-0.510 ± 0.192	0.02	-0.002 ± 0.200	1.0		
Preoperative PaO_2_	0.517 ± 0.225	0.03	0.194 ± 0. 193	0.3		
Follow-up SGOI	-0.300 ± 0.222	0.2				
Δ SGOI	0.055 ± 0.23	0.8				
CI	0.152 ± 0.221	0.5				
%Δ PVR	0.613 ± 0.177	0.002	0.338 ± 0. 179	0.08	0.360 ± 0.168	0.045*
**Obstruction ratio**	**-0.693** ± **0.159**	**0.0003**	**-0.462** ± **0. 197**	**0.03**	**-0.521** ± **0.167**	0.006*
Follow-up %VC	0.318 ± 0.212	0.1				
Follow-up FEV_1.0_/FVC%	0.088 ± 0.22	0.7				
Follow-up %DLCO/V_A_	0.415 ± 0.203	0.055				

β = standardized regression coefficient ± standard error.

*Adjusted R^2^ = 0.545, p = 0.0002, See Table 1 for abbreviations.

### Association between pulmonary arteriopathy, A-aDO_2_, and diffusion capacity after PEA

**Fig 4:**
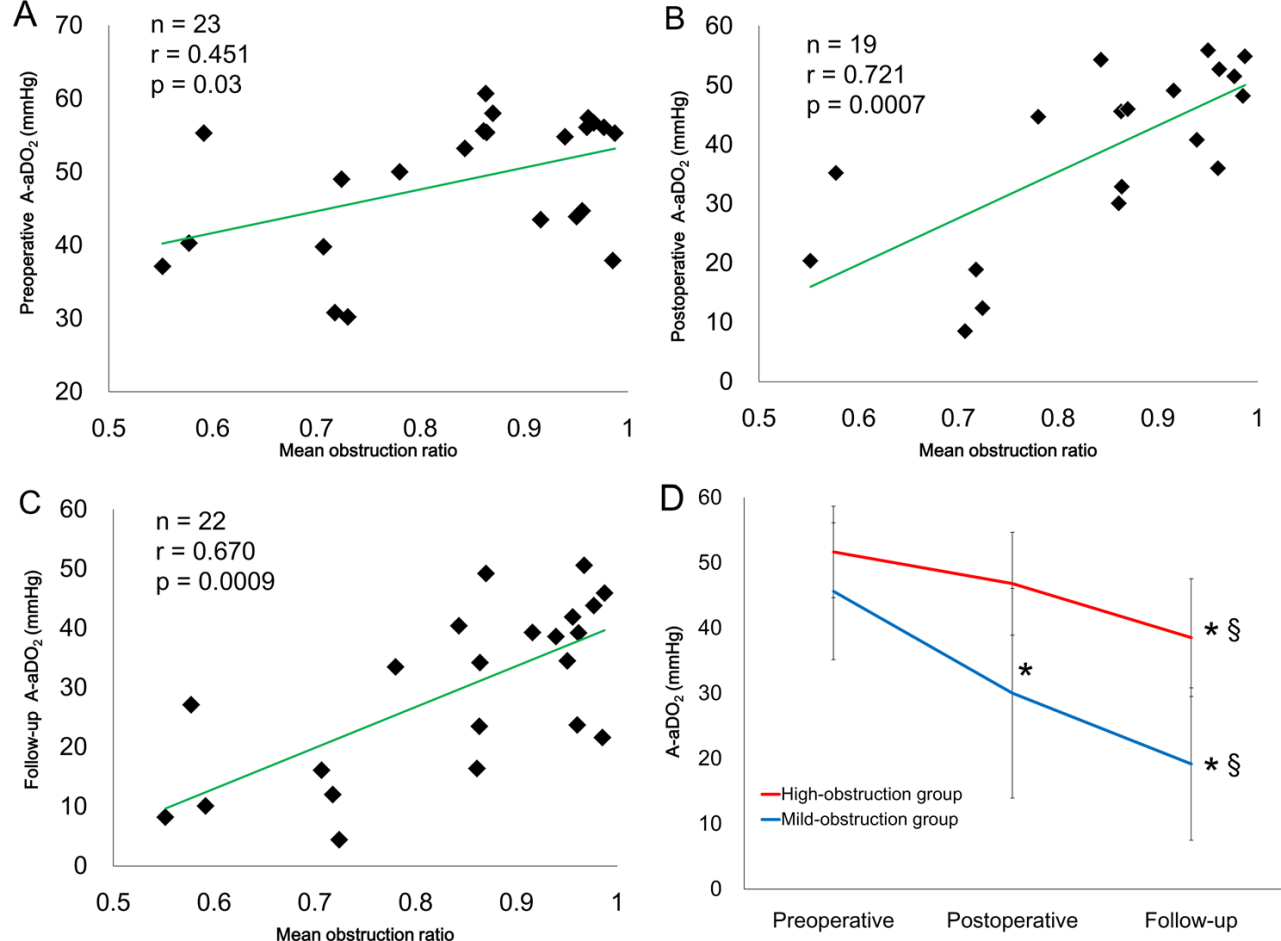
Relationship between mean obstruction ratio and A-aDO_2_. The mean obstruction ratio was negatively correlated with the preoperative (A), postoperative (B), and follow-up A-aDO_2_ (C). The high obstruction group had higher A-aDO_2_ values than the low-obstruction group (p = 0.004, analyzed by repeated measures ANOVA) (D).

**Fig 5:**
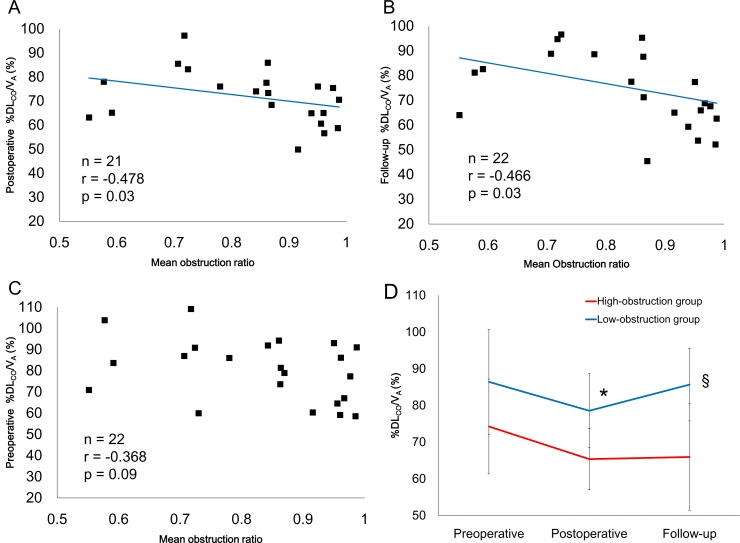
Relationship between the mean obstruction ratio and %DL_CO_/V_A_. The mean obstruction ratio was negatively correlated with postoperative (A) and follow-up %DL_CO_/V_A_ (B). However, the mean obstruction ratio did not correlate with preoperative %DL_CO_/V_A_ (C). The high obstruction group had lower %DL_CO_/V_A_ values than the low-obstruction group (p = 0.002, analyzed by repeated measures ANOVA) (D). (*: p<0.05, vs preoperative data for each group; §: p<0.05, vs postoperative data for each group).

The mean obstruction ratio positively correlated with A-aDO_2_ at all time points (Fig 4A, 4B and 4C). The high-obstruction group had a higher A-aDO_2_ than the low-obstruction group (p = 0.004, analyzed by repeated measures ANOVA, Fig 4D). The mean obstruction ratio negatively correlated with the postoperative and follow-up %DL_CO_/V_A_ (Fig 5A and 5B), but did not correlate with the preoperative %DL_CO_/V_A_ (Fig 5C). The %DL_CO_/V_A_ in the high-obstruction group was significantly lower than the low-obstruction group (p = 0.002, analyzed by repeated measures ANOVA, Fig 5D).

### Potential effect of PvO_2_ on PaO_2_ in CTEPH

PvO_2_ was positively correlated with PaO_2_ at all time points (preoperative: r = 0.425, p = 0.04; postoperative: r = 0.773, p = 0.0001; follow-up: r = 0.836, p = 0.000001).

## Discussion

In this study, we observed that severe pulmonary arteriopathy correlates with persistent hypoxemia after PEA. The extent of pulmonary arteriopathy was negatively correlated with postoperative and follow-up PaO_2_ and was an independent determinant of PaO_2_ after PEA.

We found that severe pulmonary arteriopathy is associated with persistent hypoxemia following PEA in patients with CTEPH. Hypoxemia can be caused by four mechanisms: a ventilation-perfusion mismatch, impaired diffusion capacity, intrapulmonary shunts and hypoventilation [17]. Kapitan and colleagues reported that the ventilation-perfusion mismatch, measured by gas elimination methods, could be a major contributor to hypoxemia in postoperative CTEPH patients [11]. In our study, the mean obstruction ratio positively correlated with A-aDO_2_, and patients with high obstruction ratios had little improvement in A-aDO_2_ after PEA (Fig 4). Hence, severe pulmonary arteriopathy could be related to higher A-aDO_2_ after PEA. A-aDO_2_ reflects the degree of ventilation-perfusion mismatch [13]; therefore, severe pulmonary arteriopathy might be associated with unresolved ventilation-perfusion mismatch in the pulmonary microcirculation after PEA. An impaired diffusion capacity was also associated with hypoxemia [13,17]. Low DL_CO_ results from a reduction in the pulmonary capillary blood volume (Vc) and/or pulmonary membrane diffusion capacity (D_M_) [13]. Bernstein et al. reported that Vc and D_M_ in preoperative CTEPH patients were significantly lower than in healthy control subjects and the values did not improve after PEA [18]. It was suggested that the reduction in Vc and D_M_ might be due to the pulmonary arteriopathy [18,19], and our results support this idea (Fig 5). Thus, the severe pulmonary arteriopathy in some postoperative CTEPH patients might be associated with ventilation-perfusion mismatch and impaired diffusion capacity.

The adverse effects of PEA, or other as-yet unidentified postoperative factors, might be involved in the delayed recovery of PaO_2_ one month after PEA [7,20]. In general, the VC value, which could induce hypoventilation, was depressed at one to three months following thoracotomy [21] and recovery of the respiratory function took three or more months following PEA [7]. The vascular steal phenomenon, defined as a disproportionate increase of blood flow to newly perfused lung regions [22], is a major contributor to ventilation perfusion mismatch and hypoxemia during the early postoperative period [20]. An anatomical shunt between the pulmonary and bronchial circulations develops in CTEPH patients [23,24]. Increased blood flow supplied from the two circulations [25] has been shown to induce endothelial damage, resulting in lung edema and hypoxemia in the postoperative period [26]. Intrapulmonary shunt may cause hypoxemia in CTEPH patients; however, current data are contradictory. It has been reported that anatomical shunts between the pulmonary arteries and veins (PA-PV shunt) are present in CTEPH patients [24]. Tanabe et al. reported that preoperative CTEPH patients had an increased absolute shunt ratio, and this did not decrease after PEA [20]. In the present study, the preoperative shunt ratio by perfusion scans was slightly increased over the normal range (8%), and the values after PEA were evaluated in a few patients (data not shown). However, Kapitan et al. found that intrapulmonary shunts do not exist in this patient population and are not responsible for hypoxemia in CTEPH patients [10,11]. The reason for these discrepancies may be that different methods were used in the studies [10,11,20].

There is a discrepancy in the prevalence of hypoxemia and residual PH following PEA in patients with CTEPH [7,8]. The precise mechanisms underlying this inconsistency remain unknown. Some patients demonstrate an increased shunt ratio on preoperative perfusion scans. The preoperative shunt ratio weakly correlated with PaO_2_ at follow-up (r = -0.502, p = 0.02), but did not correlate with preoperative PaO_2_ (r = -0.299, p = 0.08) (data not shown). The shunt ratio data following PEA were too sparse for robust analysis, but our group previously reported that the shunt ratio did not change after PEA [20]. Severe vascular remodeling might contribute to persistent shunting, resulting in inconsistent shunt observations in this patient population.

**Fig 6:**
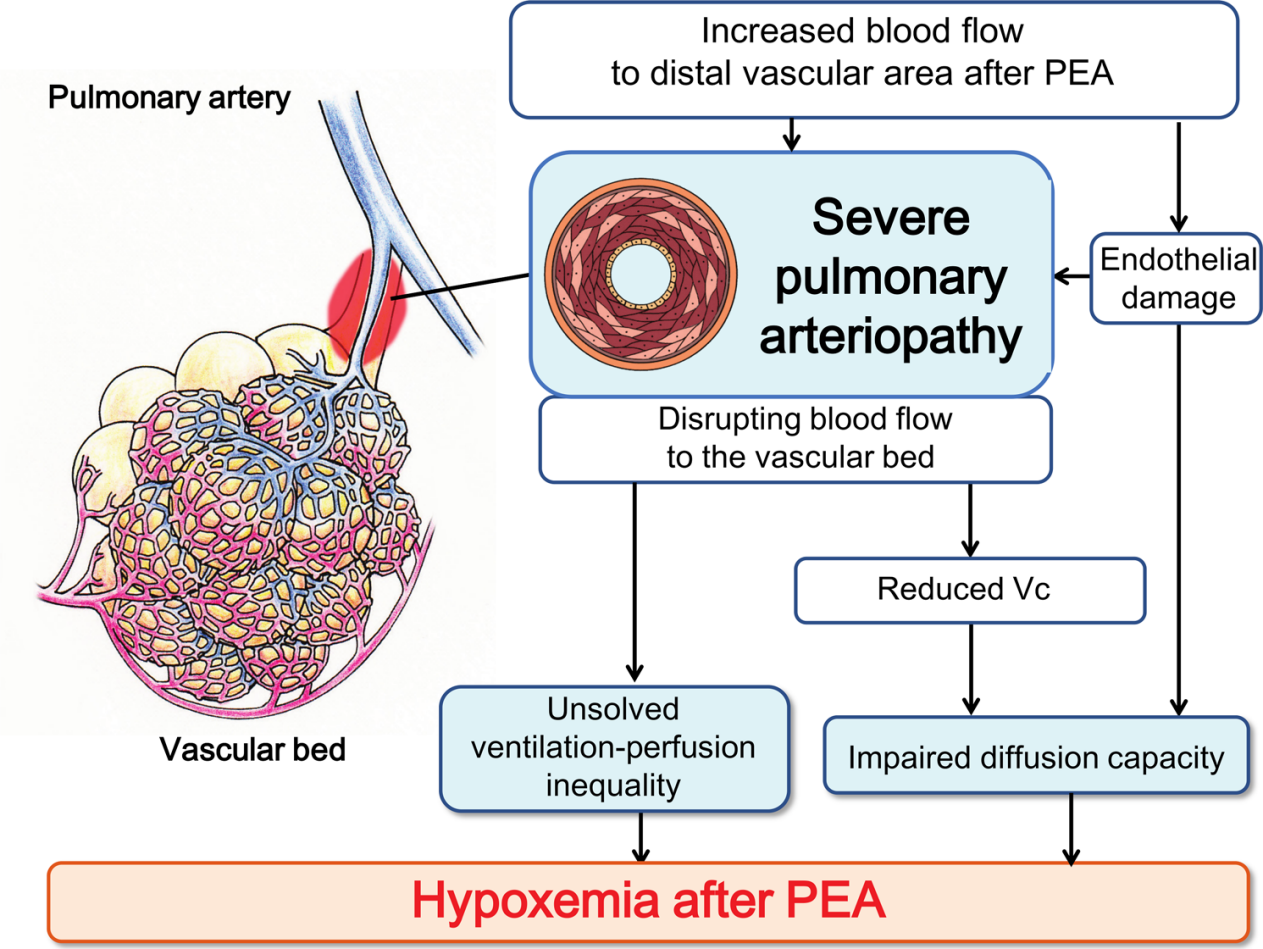
The effects of severe pulmonary arteriopathy on oxygenation and hemodynamics following PEA in patients with CTEPH. Severe pulmonary arteriopathy might negatively influence the pulmonary microcirculation, resulting in residual hypoxemia after PEA. Vc: pulmonary capillary blood volume.

Fig 6 illustrates our hypothesis that severe pulmonary arteriopathy contributes to persistent hypoxemia after PEA. Removal of chronic segmental pulmonary thrombi by PEA would be expected to increase blood flow to distal vascular areas, but persistent pulmonary arteriopathy would cause a chronic disruption of blood flow into the pulmonary capillary bed. This disruption could result in a sustained ventilation-perfusion mismatch, reduced Vc, and impaired diffusion capacity. We hypothesize that obstruction of blood flow causes a persistent intrapulmonary shunt. Hypoxemia can be sustained even one year after PEA. The persistent nature of hypoxemia may be due to an irreversible component of the pulmonary arteriopathy. It has been reported that severe pulmonary vascular remodeling persists after epoprostenol therapy [27] and successful balloon pulmonary angioplasty [28]. Reversal of pulmonary artery remodeling might improve both hypoxemia and residual PH and is a goal for future CTEPH treatment.　

The mechanisms that contribute to poor oxygenation in preoperative CTEPH patients appear to be more complicated. Ventilation-perfusion mismatch could be responsible for preoperative hypoxemia, which was amplified by the low PvO_2_ and the low cardiac output [10]. Kim et al. described two components, segmental arteries and distal vessels, that affect the pulmonary circulation in preoperative CTEPH patients [29]. It has also been reported that the extent of chronic segmental thrombi, estimated by pulmonary angiography, has minimal association with the ventilation-perfusion mismatch [10], which is in agreement with our data. In our study, the mean obstruction ratio weakly correlated with the preoperative A-aDO_2_ (Fig 4A), although the relationship between the mean obstruction ratio and the preoperative PaO_2_ was inconsistent (Fig 2C and S2 Table). Hence, multiple interrelated factors are likely to affect gas exchange in CTEPH patients prior to PEA, and the pulmonary arteriopathy might be a factor. Further investigation is needed to clarify these factors.

Pulmonary vasodilators might affect the gas exchange in CTEPH patients. Several pulmonary vasodilators were administered to some patients (S1 and S3 Tables). Riociguat was used to treat only one patient because it was not yet approved in Japan when this study began. Patients who required the pulmonary vasodilators had higher pulmonary pressure and PVR, which might be related to severe pulmonary arteriopathy [12]. All patients tended to have improved PaO_2_ between the postoperative and follow-up periods. It was difficult to examine the beneficial or harmful effects of vasodilators on gas exchange as well as vascular remodeling for postoperative patients. Some vasodilators, such as endothelin receptor antagonists and epoprostenol, might potentially worsen the gas exchange due to deteriorating ventilation-perfusion inequality [30,31], although it was reported that riociguat and bosentan did not induce hypoxemia in the clinical trials for CTEPH patients [32,33]. The influence of vasodilators on gas exchange was unclear in this study. Further investigation is needed regarding this point.

In this study, the biopsied sample size was small and only a single lung specimen was resected and examined from each patient due to safety and ethical reason. Dorfmüller et al. found evidence of pulmonary arteriopathy in 10 lung specimens randomly selected from whole lung tissue samples from each of 17 CTEPH cases [24]. Pulmonary arteriopathy has also been observed in both open and occluded vascular areas [34,35]. These results may suggest that the pulmonary arteriopathy is distributed throughout the entire lung. Finally, Yamaki’s report and our previous study indicate that the extent of pulmonary arteriopathy in a single biopsy specimen correlates with both the hemodynamics and the prognosis in operative CTEPH patients [12,36]. It was proposed that the entire pulmonary circulation might not be fully reflected in the small lung tissue samples. However, the single lung biopsy was interpreted as a random sampling of pulmonary arteries from each patient, and the specimens could provide important information regarding the pulmonary microcirculation. As an alternative to lung biopsy, the noninvasive methods of directly evaluating the pulmonary microcirculation were needed.

This study had several limitations. First, the study was performed at a single institution in Japan. Second, the small lung tissue samples might not fully reflect the entire pulmonary circulation as described above. Third, the vascular steal phenomenon was not investigated because perfusion scans were, in most cases, performed only at the time of diagnosis. Fourth, the perfusion/ventilation mismatch was not evaluated by the gas elimination method. Fifth, the chronic segmental thrombi were only evaluated by CT scan images. Finally, the relationship between vasodilators and oxygenation was not investigated.

## Conclusion

The severity of pulmonary arteriopathy was closely associated with PaO_2_ after PEA and was an independent determinant of postoperative and follow-up hypoxemia.

## Supporting Information

S1 FigIllustration detailing the quantification of pulmonary arteriopathy.The obstruction ratio of each pulmonary artery was defined as the ratio of the luminal area to the vascular area of the artery. The vascular and luminal areas were defined as the areas enclosed by the external elastic lamina and the luminal wall, respectively. The area was traced and measured using Image J software (ver. 1.45). The obstruction ratio of each pulmonary artery was calculated according to the following formula:
$Obstruction\;ratio=\frac{Vascular\;area-Luminal\;area}{Vascular\;area}$(TIF)

S1 Table Details of vasodilator medical therapy. (DOCX)

S2 Table Univariate analysis of variables associated with preoperative PaO_2_. (DOCX)

S3 Table Details of vasodilator medical therapy in the high- and low-obstruction groups. (DOCX)
