# Supplementary figures and images for: Severe Pulmonary Arteriopathy Is Associated with Persistent Hypoxemia after Pulmonary Endarterectomy in Chronic Thromboembolic Pulmonary Hypertension
Source: PLoS One. 2016 Aug 29;11(8):e0161827. doi: 10.1371/journal.pone.0161827 (PMC5003341; doi:10.1371/journal.pone.0161827)

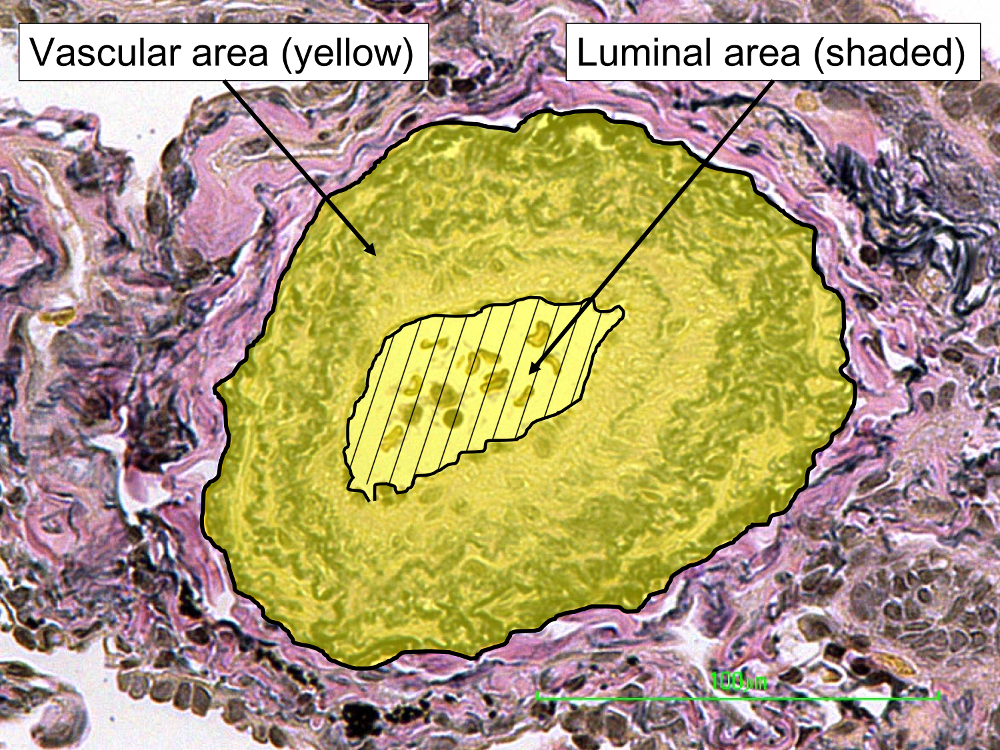

Supplement: S1 Fig — The obstruction ratio of each pulmonary artery was defined as the ratio of the luminal area to the vascular area of the artery. The vascular and luminal areas were defined as the areas enclosed by the external elastic lamina and the luminal wall, respectively. The area was traced and measured using Image J software (ver. 1.45). The obstruction ratio of each pulmonary artery was calculated according to the following formula: Obstructionratio=Vasculararea−LuminalareaVasculararea (TIF) [file pone.0161827.s001.tif]
