# Supplementary material for: Severe Pulmonary Arteriopathy Is Associated with Persistent Hypoxemia after Pulmonary Endarterectomy in Chronic Thromboembolic Pulmonary Hypertension
Source: PLoS One. 2016 Aug 29;11(8):e0161827. doi: 10.1371/journal.pone.0161827 (PMC5003341; doi:10.1371/journal.pone.0161827)
Supplement: S2 Table — (DOCX) [file pone.0161827.s003.docx]

**Supporting information**

**Title**

**Severe pulmonary arteriopathy is associated with persistent hypoxemia after pulmonary endarterectomy in chronic thromboembolic pulmonary hypertension**

**Authors**

Takayuki Jujo, Nobuhiro Tanabe, Seiichiro Sakao, Hatsue Ishibashi-Ueda, Keiichi Ishida, Akira Naito, Fumiaki Kato, Takao Takeuchi, Ayumi Sekine, Rintaro Nishimura, Toshihiko Sugiura, Ayako Shigeta, Masahisa Masuda, Koichiro Tatsumi.

**S2 Table. Univariate analysis of variables associated with preoperative PaO_2_**

|  | Simple regression analysis | |
| --- | --- | --- |
|  | β | p-value |
| Age | -0.404 ± 0.200 | 0.056 |
| SGOI | -0.087 ± 0.217 | 0.7 |
| CI | -0.270 ± 0.210 | 0.2 |
| Obstruction ratio | -0.426 ± 0.197 | 0.04* |
| %VC | 0.349 ± 0.205 | 0.1 |
| FEV_1.0_/FVC% | -0.086 ± 0.217 | 0.7 |
| %DLCO/V_A_ | -0.188 ± 0.224 | 0.4 |

* Adjusted R-squared:0.1421, p=0.04
