# Supplementary material for: Severe Pulmonary Arteriopathy Is Associated with Persistent Hypoxemia after Pulmonary Endarterectomy in Chronic Thromboembolic Pulmonary Hypertension
Source: PLoS One. 2016 Aug 29;11(8):e0161827. doi: 10.1371/journal.pone.0161827 (PMC5003341; doi:10.1371/journal.pone.0161827)
Supplement: S3 Table — (DOCX) [file pone.0161827.s004.docx]

**Supporting information**

**Title**

**Severe pulmonary arteriopathy is associated with persistent hypoxemia after pulmonary endarterectomy in chronic thromboembolic pulmonary hypertension**

**Authors**

Takayuki Jujo, Nobuhiro Tanabe, Seiichiro Sakao, Hatsue Ishibashi-Ueda, Keiichi Ishida, Akira Naito, Fumiaki Kato, Takao Takeuchi, Ayumi Sekine, Rintaro Nishimura, Toshihiko Sugiura, Ayako Shigeta, Masahisa Masuda, Koichiro Tatsumi.

**S3 Table. Details of vasodilator medical therapy in the high- and low-obstruction groups**

|  | **High-obstruction group** | | | **Low-obstruction group** | | |
| --- | --- | --- | --- | --- | --- | --- |
|  | Preoperative | Postoperative | Follow-up | Preoperative | Postoperative | Follow-up |
| **None** | **2** | **9** | **5** | **7** | **10** | **10** |
| **Monotherapy** | **4** | **3** | **5** | **4** | **0** | **0** |
| Sildenafil | 2 | 3 | 2 | 0 | 0 | 0 |
| Tadarafil | 0 | 0 | 1 | 1 | 0 | 0 |
| Bosentan | 0 | 0 | 1 | 0 | 0 | 0 |
| Beraprost | 2 | 0 | 1 | 3 | 0 | 0 |
| **Combination therapy** | **6** | **0** | **2** | **0** | **0** | **0** |
| Riociguat + Bosentan | 1 | 0 | 0 | 0 | 0 | 0 |
| Sildenafil + Bosentan | 0 | 0 | 2 | 0 | 0 | 0 |
| Tadarafil + Ambrisentan | 1 | 0 | 0 | 0 | 0 | 0 |
| Sildenafil + Beraprost | 2 | 0 | 0 | 0 | 0 | 0 |
| Bosentan + Beraprost | 2 | 0 | 0 | 0 | 0 | 0 |
